# Cytomegalovirus seroprevalence, recurrence, and antibody levels: Associations with cadmium and lead exposures in the general United States population

**DOI:** 10.1097/EE9.0000000000000100

**Published:** 2020-06-05

**Authors:** Catherine M. Bulka, Paige A. Bommarito, Allison E. Aiello, Rebecca C. Fry

**Affiliations:** a Department of Environmental Sciences and Engineering, Gillings School of Global Public Health, University of North Carolina, Chapel Hill, North Carolina; b Department of Epidemiology, Gillings School of Public Health, University of North Carolina, Chapel Hill, North Carolina; c Carolina Population Center, University of North Carolina, Chapel Hill, North Carolina; d Institute for Environmental Health Solutions, Gillings School of Global Public Health, University of North Carolina, Chapel Hill, North Carolina; e Curriculum in Toxicology, School of Medicine, University of North Carolina at Chapel Hill, Chapel Hill, North Carolina.

**Keywords:** Toxic metals, Immune system, Cytomegalovirus, Antibodies

## Abstract

The ubiquitous metals cadmium and lead are immunotoxic, but little is known about their relations to cytomegalovirus (CMV), a widespread herpesvirus. Although CMV infections are mostly asymptomatic, congenital infections are a leading cause of birth defects. In otherwise healthy individuals, there is also some evidence linking subclinical reactivations to accelerated age-related declines in immune function and chronic disease.

**Methods:**

Our objective was to evaluate associations of blood cadmium and lead biomarkers with CMV infection in a representative sample of the United States population. In seropositive individuals, we also examined associations with CMV-specific immunoglobulin G (IgG) antibody levels and suspected CMV recurrences. Using cross-sectional data from the 1999–2004 National Health and Nutrition Examination Surveys, we fit multivariable survey-weighted regression models accounting for potential confounding by sociodemographic and lifestyle factors and stratifying by age group to allow for heterogeneity. CMV recurrences were defined according to (1) the presence of either CMV-specific immunoglobulin M in sera or CMV viral DNA in urine, and (2) high CMV-specific IgG avidity.

**Results:**

We observed null associations for blood cadmium. Increasing blood lead quartiles were related to CMV seropositivity and higher CMV IgG levels (both *P*_*trend*_ < 0.01), but not CMV recurrence, only among individuals who were 20–29 years of age.

**Conclusion:**

Blood cadmium levels do not appear to be related to immunological markers of CMV infections. The possibility that lead exposures increase the risk of CMV infection and impair immune control of the virus in young adults was suggested. Prospective studies are needed to confirm.

## Abstract 2

Supplemental Digital Content is available in the text.

**What this study adds**

Cytomegalovirus (CMV) is extremely common and emerging evidence suggests latent infections could pose serious health risks by impairing immune function. Yet, very little is known about environmental risk factors for CMV. Prior studies have linked cadmium and lead exposures to altered innate and adaptive immune responses, but no studies to date have specifically examined these toxic metals in relation to persistent and immune modulated infections such as CMV. To address this gap, we analyzed a representative sample of the United States population with blood measurements of cadmium and lead and evaluated associations with CMV seroprevalence. Among individuals considered to have latent infections, we additionally investigated metal associations with infection recurrences and antibody levels as these may be indicators of poor immune control of the virus.

## Introduction

Cytomegalovirus (CMV), a member of the herpesvirus family, is one of the most common viruses globally and nationally with approximately half of the United States population infected.^1,2^ The virus is mostly asymptomatic in healthy individuals, but some populations are at heightened risk for substantial morbidity and mortality.^3^ Among newborns, for instance, congenital infections are a leading cause of permanent hearing loss, visual impairments, mental retardation, and even death.^3^ Without a vaccine currently available, identifying modifiable risk factors for CMV infection is a public health priority.^4^ Although preventing contact with infected bodily fluids is the most effective strategy for reducing CMV transmission, reducing exposure to environmental factors might also play a role.^5,6^ The environmentally pervasive toxic metals cadmium and lead represent biologically plausible putative risk factors for the virus as epidemiologic studies suggest these contaminants affect both innate and adaptive immunity by altering amounts of monocytes, eosinophils, neutrophils, and basophils, as well as natural killer and T helper cells.^7–9^ Data on the infectious disease consequences of cadmium and lead exposures are limited, although these metals have been cross-sectionally associated with chronic infections by *Helicobacter pylori* bacteria, *Toxoplasma gondii* parasites, and the hepatitis B virus.^10^ However, the relationship between toxic metals and CMV infection has yet to be evaluated.

Acute CMV infections are rarely serious for immunocompetent individuals, but among those otherwise considered healthy, accumulating evidence links the virus to adverse health effects. Like all herpesviruses, CMV persists in the body for life entering a latency period following primary infection.^11^ Hence, the immune system is continuously working towards keeping the virus in a quiescent state.^11^ Despite this constant surveillance, reinfections by a different strain or reactivations occur. Such recurrences are posited to impair the adaptive immune response in a manner similar to the natural aging process.^12–14^ Specifically, although the causal link remains a matter of debate, CMV infection appears to increase the number of memory T cells at the expense of naïve T cells, ultimately limiting one’s ability to respond to new pathogens.^15,16^ CMV infections have also been prospectively associated with systemic inflammation as well as cardiovascular disease morbidity and mortality.^17–22^ Immune system alterations from repeated reinfections and reactivations of CMV infection over the life course may be an important mechanism for chronic disease development.

Distinguishing recurrent from primary CMV infections is challenging due to the need for laboratory assays with high specificity. Following the initial infection, the host begins to produce immunoglobulin G (IgG) and immunoglobulin M (IgM) antibodies specific to the virus within 1–2 weeks.^23^ The production of CMV-specific IgG will continue for life, thus, their presence in sera serves only as an indicator of past exposure to the virus. In contrast, the production of CMV IgM tends to halt 3–6 months after the primary infection but can restart when reinfected by a different viral strain or when latent infections are reactivated.^24^ Additional methods for identifying active CMV infections are through viral culture or polymerase chain reaction (PCR) because, in order for CMV to be transmitted to a new host, viral replication is accompanied by shedding in bodily fluids.^25^ However, neither the presence of IgM in sera nor the detection of CMV in bodily fluids provides adequate information to differentiate between primary and non-primary infections. Due to these limitations, the avidity of CMV IgG has been proposed as a superior measure. Avidity refers to the strength with which an antibody binds to an antigen.^24^ Avidity matures over time as B cells that produce IgG that bind more tightly are gradually selected.^24^ For the first few months following primary infection, IgG binds weakly to CMV thus exhibiting low avidity.^24^ About 6 months after initially encountering CMV, the produced IgG exhibit high avidity.^24^

Perhaps because of the need for multiple serologic measures to identify recurrent CMV infections, levels of CMV IgG are increasingly being used as a surrogate. Although high levels can indicate a recent primary infection, the young age at which the immune system typically first encounters the persistent virus means they could alternatively reflect increased antibody production to counteract reactivation.^26–30^ In support of this premise, studies have demonstrated positive correlations for CMV IgG levels with urinary shedding of the virus, number of viral DNA copies found in blood, and frequency and duration of reactivations.^31–33^ Others have consistently observed positive associations for socioeconomic disadvantage and psychosocial stress with higher CMV IgG levels.^34–37^ These findings suggest exposures to certain stressors could impair immune control of CMV and provide it with opportunities for replication and circulation.^38^ Environmental toxicants have also been linked to CMV IgG levels with cross-sectional associations observed for bisphenol A and triclosan.^6^ Yet, there are no studies examining the association between toxic metal exposures and CMV. Given the ubiquity of cadmium and lead in cigarette smoke, contaminated drinking water, and some foods (e.g., grains, vegetables, and fruits) in conjunction with their potential for immunotoxicity, more research on the interplay between environmental exposures to toxic metals and infectious disease is needed.^39,40^ In the present analysis, we sought to determine whether cadmium and lead are related to CMV infection and immune response within the general population of the United States. We hypothesized that greater exposures to toxic metal would be associated with CMV seropositivity and that, among seropositive individuals, higher cadmium and lead levels correlate with CMV recurrence and higher CMV IgG antibody levels.

## Methods

### Study population

**Figure 1.**
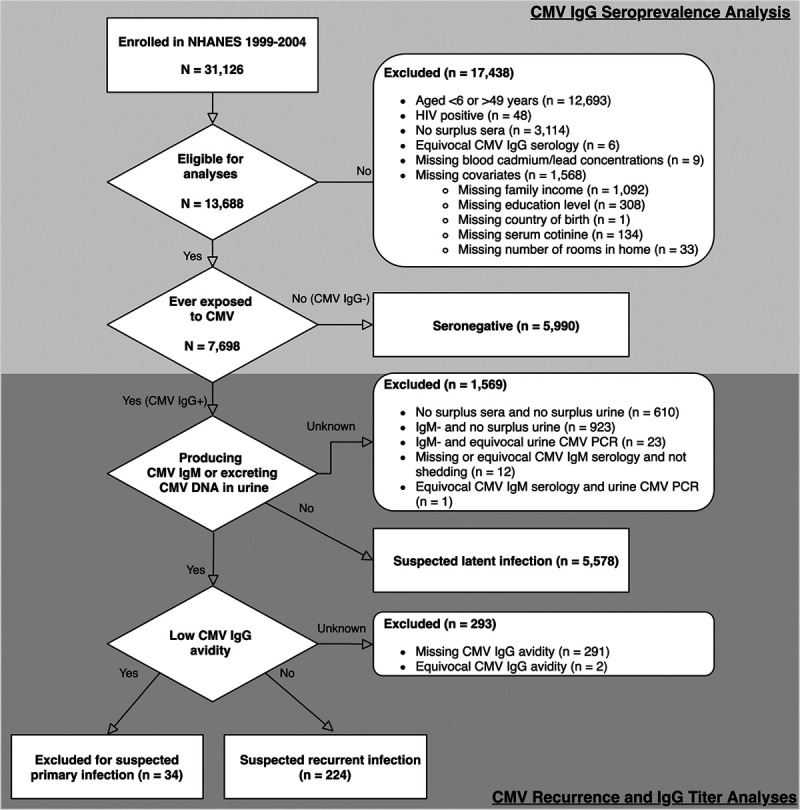
Inclusion/exclusion criteria.

The National Health and Nutrition Examination Survey (NHANES) is a series of nationally-representative cross-sectional surveys of the non-institutionalized United States population conducted using complex, multi-stage probability sampling by the National Center for Health Statistics. Study procedures were approved by the National Center for Health Statistics Research Ethics Review Board (Protocol #98-12) with all participants providing documented consent. During 1999–2004, 31,126 participants underwent in-person interviews and physical examinations. Our analyses focused on participants with blood measurements of lead and cadmium, serum CMV IgG, and complete data on relevant covariates (Figure 1). We excluded participants who tested positive for HIV due to their compromised immune status (n = 48). Although all study participants older than 1 year were eligible for the measurement of blood cadmium and lead levels, CMV-specific antibody testing was only performed among participants aged 6 to 49 years with sufficient surplus sera (n = 15,316). In addition, quantification of CMV DNA in urine was limited to IgG-positive participants who were either non-Hispanic white, non-Hispanic black, or Mexican American and had surplus urine specimens available (n = 6859). The final analytic sample sizes were comprised of 13,688 individuals for analyses of CMV IgG seroprevalence and 5,802 individuals (224 individuals suspected to have a recurrent infection and 5,578 suspected to have a latent one) for analyses of CMV recurrence and CMV IgG antibody levels.

### Metal biomarkers assessment

Participants were requested to provide venous blood samples in the mobile examination center. Specimens were shipped on dry ice to the National Center for Environmental Health in Atlanta, GA. For participants enrolled in the 1999–2002 surveys, blood cadmium and lead concentrations were simultaneously measured by electrothermal atomic-absorption spectrophotometry, as described elsewhere.^10^ For participants enrolled in the 2003–2004 survey, blood cadmium and lead were determined by inductively coupled plasma dynamic reaction cell-mass spectrometry.^41^ The lower limits of detection (LOD) for cadmium were 0.3 μg/L during 1999–2002 and 0.2 μg/L during 2003–2004. For lead, the LOD remained 0.3 μg/dl throughout 1999–2004. The majority of individuals had detectable levels of cadmium (57.9%) and lead (99.0%) in their blood. As a sensitivity analysis, we also evaluated cadmium concentrations in urine, which were detected more frequently (93.2%, see Sensitivity Analysis section for more details).

### Cytomegalovirus serology

The primary endpoint of interest was the presence of CMV IgG as measured in sera. All serologic testing was conducted by trained laboratory personnel at the Centers for Disease Control and Prevention in Atlanta, GA. CMV IgG was measured in stored surplus sera specimens using an enzyme-linked immunosorbent assay (ELISA) developed by Quest International, Inc. (Miami, Florida). Approximately 5.2% of samples were found to have equivocal results near the cutoff value. Thus, a second ELISA (bioMerieux, Inc., Durham, North Carolina) was performed with any concordant positive or negative results reported as such. A remaining 2.7% of the samples were discordant, so confirmatory testing was performed using an immunofluorescent assay (Bion International, Inc., Des Plaines, Illinois). CMV IgG status was qualitatively reported as seronegative or seropositive. After the immunofluorescent assay, a small number of samples (n = 6, Figure 1) remained equivocal, and were excluded from subsequent analyses.

Secondary endpoints of interest included CMV recurrence and CMV IgG antibody levels. Quantitative CMV-specific IgG optical density measures from the first ELISA (Quest International, Inc.) were reported. Levels were measured in arbitrary units per milliliters (AU/ml) with values above the maximum detectable threshold of 3.00 AU/ml top-coded.^42^ Among CMV IgG seropositive individuals, screening for CMV IgM was performed using an ELISA by Diamedix (Miami Lakes, Florida) and the automated analyzer MAGO. Specimens with optical density ratios <0.90 were considered seronegative and those ≥1.10 were considered seropositive. Indeterminate specimens between ≥0.90 and <1.10 were omitted from our analyses (Figure 1). Confirmatory IgM testing was performed on specimens near the test cutoff values using the Vidas ELISA test (bioMerieux, Inc.) with results interpreted according to the manufacturer’s instructions. IgG avidity was measured by performing two CMV-specific IgG tests in parallel using the Vidas ELISA (bioMerieux, Inc.) One test was used for reference IgG determination. For the second test, 6 M urea solution was added to the wash buffer as a protein denaturing agent in order to weaken the antigen-antibody bond.^43^ Avidity indexes were calculated by dividing the optical density with urea by the optical density of the reference sample with values ≥0.8 reported as high avidity (suggestive of past infection), >0.7 and <0.8 reported as indeterminate, and <0.7 reported as low avidity (suggestive of recent infection).

### Cytomegalovirus shedding

Stored surplus urine specimens from CMV IgG seropositive non-Hispanic white, non-Hispanic black, and Mexican American participants were tested for the occurrence of viral shedding.^44^ Viral DNA was extracted with the QIAmp MinElute Media Kit and processed with the Qiacube automated extractor (both manufactured by Qiagen, Valencia, California). Detection of CMV DNA was performed by a real-time PCR assay that targeted the highly conserved immediate-early 2 exon 5 region using the MX 3005P Real-time PCR System (Agilent Technologies, New Castle, Delaware).^45^ The lower limit of detection was 80 copies/ml, equivalent to 80 international units per milliliter (SC Dollard, written communication, May 2019). PCR testing was performed in duplicate for all specimens, with two positive results required for specimens to be reported as positive.^44^ A summary of the CMV laboratory tests used in this analysis is provided in Table 1 of the Supplemental Digital Content; http://links.lww.com/EE/A90.

### Cytomegalovirus recurrence

In addition to evaluating CMV IgG levels as a biomarker of viral reactivation, we leveraged the multitude of CMV-specific laboratory measures available in NHANES to assess CMV recurrence more directly. To do so, we modified an algorithm originally developed to identify primary CMV infections in pregnant women to incorporate urinary shedding status along with measures of CMV IgG/IgM seropositivity and IgG avidity (Figure 1).^46^ Specifically, individuals who were IgG seropositive and also excreting CMV in their urine or seropositive for IgM were considered to have evidence of an active infection. If their IgG avidity was low, they were classified as having a primary infection presumably within the preceding 3–4 months; in contrast, individuals with high IgG avidity were classified as experiencing a recurrence.^24^

As our hypotheses for secondary analyses regarding toxic metal exposures and immune control of CMV apply only to individuals infected in the past, we excluded IgG seronegative individuals as well as those we suspected of having a primary infection (Figure 1). For a more detailed tabulation of how we classified individuals as (1) uninfected; (2) primary CMV infection; (3) latent CMV infection; or (4) recurrent CMV infection, please see Table 1.

**Table 1.**
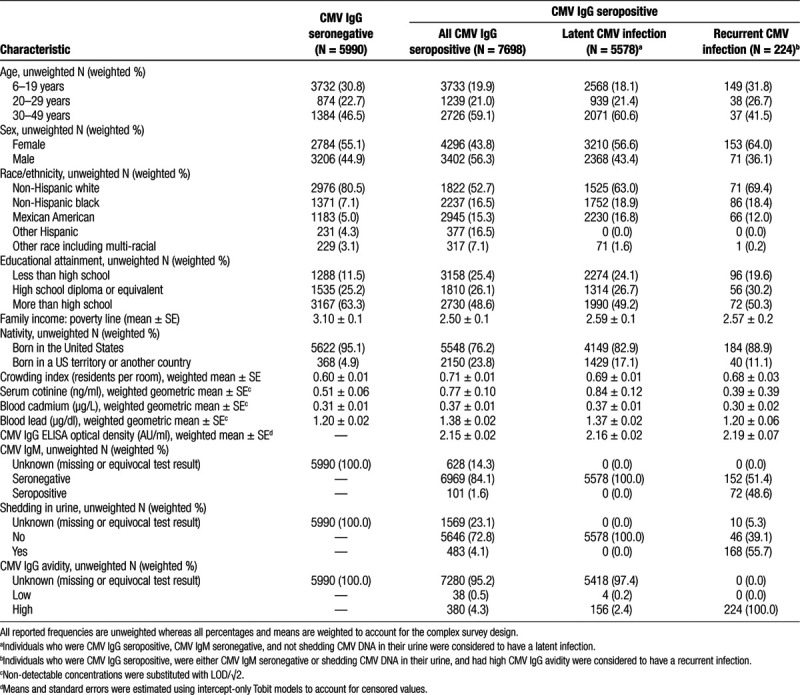
Selected population characteristics among individuals aged 6–49 years, NHANES 1999–2004.

### Sociodemographic and lifestyle characteristics

**Figure 2.**
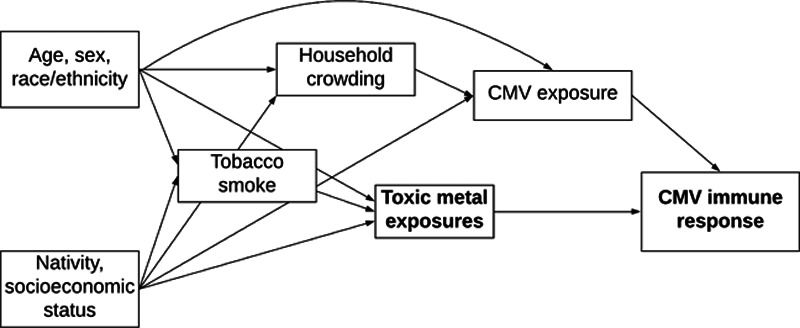
Directed acyclic graph showing the posited relationships between sociodemographic and lifestyle characteristics with toxic metal exposures and CMV. The bolded text represents the exposures (toxic metals) and outcomes (CMV seropositivity, recurrence, and IgG antibody level) of interest.

Covariates hypothesized to be potential confounders of the relations between toxic metal biomarkers and CMV infection, recurrence, and IgG levels included age, sex, race/ethnicity, socioeconomic status, nativity, and tobacco smoke exposure as these factors have either been related to toxic metal exposures and increased susceptibility to infections in general or CMV in particular.^37,44,47–49^ Household crowding was additionally studied as an independent risk factor for CMV exposure (Figure 2).^50,51^ During household interviews conducted by trained study staff, participants were asked to self-report sociodemographic and housing characteristics. Age at the time of the interview, as reported in years, was treated as a continuous variable. Race/ethnicity was categorized as non-Hispanic white, non-Hispanic black, Mexican American, other Hispanic, or other including multi-racial. Educational attainment was categorized as less than a high school diploma, high school diploma, or more than high school. For individuals below the age of 25 years, we measured the educational attainment of the household reference person (i.e., the first household member 18 years of age or older listed on the household member roster, who owns or rents the residence where household members reside). For those aged 25 years or above, educational attainment was based on the participant themselves. Family income was normalized to the poverty line and treated as a continuous variable. The resulting family income to poverty line ratio along with educational attainment was considered proxies for socioeconomic status. Nativity was defined according to the state or foreign country in which the participant was born and was categorized as within the United States (excluding territories) or elsewhere. Levels of cotinine in serum samples were measured by isotope-dilution high-performance liquid chromatography and served as an integrated biomarker of tobacco smoke exposure reflecting both active and passive exposures. The LOD for serum cotinine was 0.05 ng/ml during the 1999–2002 cycles and was reduced to 0.015 ng/ml during the 2003–2004 cycle due to laboratory improvements. For the proportion of samples with non-detectable cotinine levels (22.0%), the LOD/√2 was used. Finally, a household crowding index was derived by dividing the reported total number of people residing in the participant’s household by the number of rooms in the home.^50^

### Statistical analyses

All analyses accounted for the complex multistage probability sampling design, survey non-response, and post-stratification by incorporating survey design variables and sampling weights. NHANES assigns each sampled person a weight that quantifies the number of people in the civilian, non-institutionalized United States population represented by that one individual. However, because the present study relied on availability of surplus specimens, the sampling examination weights had to be adjusted. Briefly, since we combined three survey cycles, we first rescaled the original examination sampling weights so that their sum matched the survey population at the midpoint of 1999–2004. Next, the rescaled examination sampling weights were multiplied by the weighted proportion of available surplus samples for that participant’s sex, age, and racial/ethnic group.^50^ Standard errors were estimated with Taylor series linearization. Statistical analyses were performed with Stata/SE version 15 (College Station, Texas).

Associations of the metal biomarkers with CMV IgG seropositivity and recurrence were evaluated by fitting multivariable Poisson regression models to estimate prevalence ratios with 95% confidence intervals (CIs). For analyses of CMV IgG antibody optical densities, we used Tobit models to estimate mean differences with 95% CIs allowing for right-censoring of top-coded values.^52^ As our hypotheses for metal-associated loss of immune control of CMV only apply to individuals with persistent infections, models of CMV recurrence and IgG levels were fit only among those who were IgG seropositive and considered to have been infected in the past (see Figure 1 for details on the analytic sample breakdown).

Metal biomarker concentrations were modeled as categorical variables. Cadmium concentrations were categorized as <0.30, 0.31–0.40, 0.41–0.60, or 0.61–8.50 μg/L; these cutpoints correspond to the highest LOD across the combined NHANES cycles and the 33.3rd and 66.7th percentiles among detectable concentrations. Lead concentrations were divided into quartiles (0.20–0.80, 0.81–1.20, 1.21–1.89, and 1.90–68.90 μg/dl). Tests for linear trends were performed by refitting models with the metal category as an ordinal variable.

A prior animal study suggested viral infections might manifest differently in the presence of cadmium-lead co-exposures, thus, a cross-product term was included in the models to test for interaction.^53^ We also considered the possibility of effect modification by age since cadmium and lead bioaccumulate over time and since age is strongly tied to rates of CMV infection and antibody levels.^54–56^ We categorized age into three groups: 6–19, 20–29, and 30–49 years. In addition to unadjusted models, we fit two sequentially adjusted models to account for hypothesized confounders. Minimally adjusted models included age, sex, race/ethnicity, educational attainment, family income to poverty ratio, nativity, and serum cotinine levels. Fully adjusted models further included household crowding, a recognized risk factor for CMV infection not expected to be directly related to toxic metal exposures.^57^

### Sensitivity analysis

The proportion of blood cadmium concentrations below the LOD was large (42.1%). Therefore, we conducted a sensitivity analysis to consider cadmium measured in urine, which had a higher detection rate of 93.2%. Cadmium accumulates in the kidneys where a portion is continuously excreted in urine, thus relative to blood measures, urinary concentrations are considered reflective of longer-term exposures.^58^ In NHANES 1999–2004, urinary cadmium concentrations were measured in spot urine specimens collected from a random one-third sub-sample with inductively coupled plasma mass spectrometry. The LOD for urinary cadmium concentrations was 0.060 μg/L and concentrations below this level were assigned a value of the LOD/√2. Models of CMV IgG seropositivity were re-run substituting urinary cadmium in place of blood concentrations. Urinary creatinine was included as an additional term in these models to account for inter- and intra-individual differences in urine dilution.^59^ Due to the small sample size of CMV seropositive individuals for whom urinary cadmium, CMV serology, and CMV PCR measurements were available (n = 673 of whom 23 were suspected of having a recurrent infection), we did not carry out formal modeling of associations for urinary cadmium with CMV recurrence or IgG level.

## Results

The weighted seroprevalence of CMV IgG was 50.8% (7,698 of 13,688 individuals). Selected population characteristics are displayed by seroprevalence status in Table 2. Individuals who were seropositive for CMV IgG were older and more likely to be male, a racial/ethnic minority, lower in socioeconomic status, born in a US territory or another county, and live in more crowded households. On average, those who were CMV seropositive had higher concentrations of serum cotinine indicating more tobacco smoke exposure and had higher levels of cadmium and lead in their blood. Among individuals considered to have a latent or recurrent CMV infection, IgG antibody levels were similar at 2.16 and 2.19 AU/ml, respectively (Table 2). Of the 224 individuals suspected of experiencing a recurrent infection, CMV DNA was detected in the urine of 58.8% (Table 2).

**Table 2.**
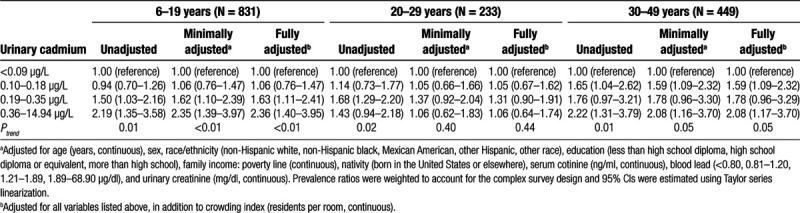
Prevalence ratios (95% CI) of CMV IgG seropositivity for urinary cadmium by age group, NHANES 1999–2004 (N = 1513)

### Blood metal concentrations with CMV IgG seroprevalence

**Figure 3.**
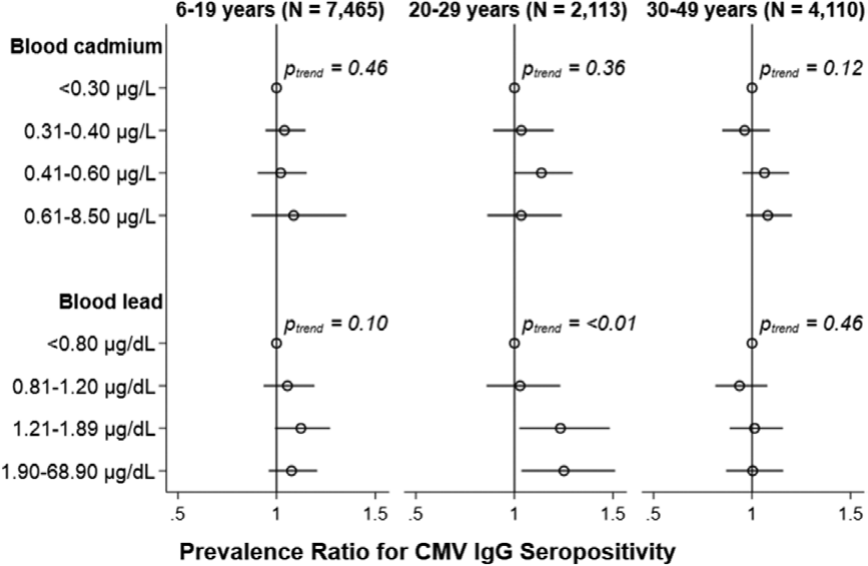
Adjusted cross-sectional associations of blood cadmium (upper) and blood lead (lower) with CMV IgG seropositivity by age group, NHANES 1999–2004. Prevalence ratios (95% CI) adjusted for age, sex, race/ethnicity, education, family income: poverty line, nativity, serum cotinine, and household crowding with mutual adjustment for blood cadmium and lead categories. Prevalence ratios were weighted to account for the complex survey design and 95% CIs were estimated using Taylor series linearization.

Adjusted Wald tests of the cross-product terms between blood cadmium and lead concentration categories were not statistically significant (all *P*_interaction_ > 0.2). However, because the metals were moderately positively correlated with one another (*r*_*s*_ = 0.30, *P*-value < 0.001), both were included in the same regressions to control for potential confounding by co-exposure. The interaction term for lead quartiles by age group was suggestive at *P*_interaction_ = 0.06 so stratified results are presented (Figure 3; see Table 2, Supplemental Digital Content; http://links.lww.com/EE/A90). After adjusting for all covariates including blood cadmium, individuals aged 20–29 years with blood lead concentrations between 1.20 and 1.89 μg/dl were 23% (95% CI = 3%, 48%) more likely to be CMV IgG seropositive whereas those with concentrations exceeding 1.89 μg/dl were 25% (95% CI = 4%, 51%) more likely to be CMV IgG seropositive relative to individuals in the same age group with blood lead concentrations below 0.80 μg/dl. However, no such associations were found among individuals aged 6–19 or 30–49 years. Point estimates for blood cadmium categories were near null across all age groups.

### Blood metal concentrations with CMV recurrence

**Figure 4.**
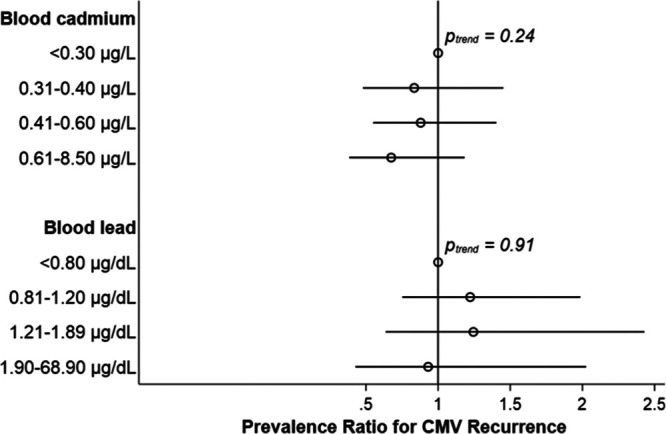
Adjusted cross-sectional associations of blood cadmium (upper) and blood lead (lower) with CMV recurrence among individuals aged 6–49 years, NHANES 1999–2004. Prevalence ratios (95% CI) adjusted for age, sex, race/ethnicity, education, family income: poverty line, nativity, serum cotinine, and household crowding with mutual adjustment for blood cadmium and lead categories. CMV recurrence was defined as being CMV IgG seropositive, being either CMV IgM seropositive or excreting CMV DNA in urine, and having low CMV IgG avidity (N = 224). Individuals who were CMV IgG seropositive but were seronegative for CMV IgM and were had no CMV DNA detected in their urine (N = 5578) served as the comparison group, as these individuals were considered latently infected. Prevalence ratios were weighted to account for the complex survey design and 95% CIs were estimated using Taylor series linearization.

We found no statistical evidence for an interaction between blood cadmium and lead levels nor for interactions between the metal biomarkers and age with respect to CMV recurrence (all *P*_interaction_ > 0.2). Thus, we present the results of models fit amongst all individuals aged 6–49 years. We observed null associations for both blood cadmium and blood lead with CMV recurrence relative to having a suspected latent infection (Figure 4; see Table 3, Supplemental Digital Content; http://links.lww.com/EE/A90).

### Blood metal concentrations with CMV IgG antibody levels

**Figure 5.**
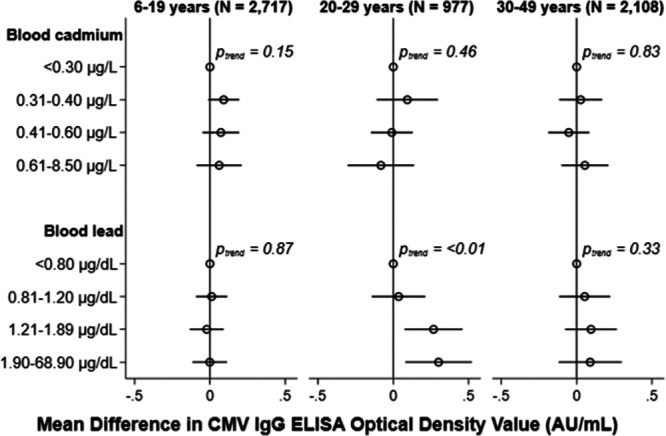
Adjusted cross-sectional associations of blood cadmium (upper) and blood lead (lower) with CMV IgG antibody levels by age group, NHANES 1999–2004. Mean differences (95% CI) adjusted for age, sex, race/ethnicity, education, family income: poverty line, nativity, serum cotinine, and household crowding with mutual adjustment for blood cadmium and lead categories. Mean differences were weighted to account for the complex survey design and 95% CIs were estimated using Taylor series linearization.

With respect to CMV IgG levels, we again did not detect a statistical interaction between the two metals (all *P*_interaction_ > 0.2). We did, however, observe a significant interaction between blood cadmium categories and age (*P*_interaction_ < 0.01). Yet, no clear dose-response trends emerged for associations of cadmium with CMV-specific IgG levels among those suspected to have a latent or recurrent infection across the three age groups (Figure 5; see Table 4, Supplemental Digital Content; http://links.lww.com/EE/A90). Meanwhile, increasing quartiles of blood lead concentrations were positively related to CMV IgG levels only in individuals aged 20–29 years (*P*_*trend*_ < 0.01). In these young adults, blood lead concentrations in the highest quartile were associated with 0.30 AU/ml (95% CI = 0.08, 0.52) higher antibody levels relative to the lowest quartile (Figure 5).

### Sensitivity analyses

Cadmium concentrations in urine were moderately correlated with levels measured in blood (*r*_*s*_ = 0.42, *P*-value = 0.001). In age-stratified models, increasing urinary cadmium concentrations were positively related to CMV IgG seropositivity. This relationship was most pronounced in children with urinary cadmium concentrations in the highest quartile who had a prevalence ratio of 2.36 (95% CI = 1.40, 3.95; Table 2).

## Discussion

In this representative sample of the United States population, we found little evidence that exposures to cadmium, as measured in blood, were related to CMV infection, antibody levels, or recurrence. For the toxic metal lead, we also found no associations with CMV parameters in children aged 6–19 years or in adults aged 30–49 years. However, in young adults aged 20–29 years, higher lead exposures were related to a higher prevalence CMV infection and higher CMV IgG levels, but not CMV recurrence. This suggests the immunotoxicity of lead is not strong enough to tip the balance towards recurrence, but could slightly impair the immune system’s capacity to contain the virus quickly when recurrences occur in young adulthood.^32^

The results of this study contribute to the growing body of literature focused on the intersection of environmental toxicant exposures and infectious disease. This analysis is the first to study toxic metal exposures in relation to CMV, yet our findings share similarities with other recent studies of NHANES data. In one such study by Krueger and Wade^10^ examining blood lead levels in relation to *H. pylori* seropositivity, the strongest associations were observed in children aged 3–12 years, followed by individuals aged 13–35 years whereas no association was found in older adults. In another NHANES analysis of CMV IgG levels, Clayton et al^6^ found an interesting pattern: higher levels of bisphenol A in urine were associated with lower levels in children aged 6–18 years but with higher levels in individuals aged 18–49 years. It is unclear why we found blood lead concentrations to be associated with CMV seropositivity and IgG levels in young adults but not at other ages; however, the aforementioned results from other NHANES analyses in combination with our results raise several possibilities. For one, younger adults may be more vulnerable to the immunotoxic effects of lead than older adults. While lead exposures were related to *H. pylori* seropositivity in children, the lack of an association with CMV may be explained by differences in the developing immune system’s response to bacteria versus persistent viruses.^10^ It is also possible that the observed associations reflect unmeasured confounding for which we were unable to fully control. More specifically, although we controlled for factors related to socioeconomic status (family income, educational attainment), these measures may not fully capture current socioeconomic status during young adulthood, a period marked by transitions and the establishment of independence.^60^ Since low socioeconomic status is related to greater cadmium and lead exposures as well as to greater CMV exposure (for instance, from daycare attendance, number of sexual partners, and crowded housing conditions),^48,56^ we would expect any residual confounding to result in overestimated associations of blood metal concentrations with CMV seropositivity and antibody levels. Finally, while elevations in CMV-specific IgG levels might correlate with the length or frequency of CMV recurrences in immunosuppressed patients,^32^ their significance in immunocompetent individuals is unclear.^61^

Analyses of cadmium as measured in blood resulted in null associations with each of the CMV parameters evaluated. However, sensitivity analyses suggested increasing levels of cadmium as measured in urine were associated with greater CMV IgG seroprevalence, particularly in children. Urinary cadmium captures longer-term exposures than blood cadmium, which may be more relevant for the studied endpoints.^62^ Children may be more susceptible to chronic cadmium exposures than adults as data from animal studies have demonstrated younger animals absorb the metal more readily than older ones.^63^ Future studies seeking to elucidate underlying mechanisms, as well as how the timing and duration of toxic metal exposures influence immune function should therefore consider measuring cadmium in urine samples.

Important strengths of this study include the use of a nationally-representative sample of the United States population, reliance on objective toxic metal biomarkers and laboratory measures of CMV, and adjustment for determinants of metal exposures and CMV risk factors. However, there are some limitations to our analyses that should be considered. The cross-sectional design of NHANES means cadmium and lead levels were measured at the same time as CMV antibodies. Therefore, we cannot discount the possibility of reverse causality. For instance, it is possible that CMV infections alter how the body stores or metabolizes toxic metals, but there are no current data to support this hypothesis. Another limitation is that, based on the characteristics of IgG, a contrary interpretation of the results may exist. A CMV-specific IgG response reflects a past or current infection and a lack thereof reflects susceptibility to the virus. Thus, the positive associations observed with CMV seropositivity in young adults could indicate cadmium and lead actually protect against CMV infection. Such an explanation is unlikely given the recognized immunotoxicity of cadmium and lead exposures and the fact that we saw a positive association between blood lead and IgG antibody levels even after excluding individuals thought to have been infected recently. However, this issue could be clarified by future prospective studies in which metal biomarkers are measured prior to repeated serologic testing allowing for the evaluation of seroconversion.^8,9,64^ Relatedly, we relied on a serological testing algorithm (modifying it to include urinary CMV shedding) to identify recurrent infections, which was not its original intent.^46^ In doing so, we may have misclassified cases, including four individuals we considered to have a latent infection (due to being CMV IgM seronegative and negative for urinary shedding) despite having low CMV IgG avidity indicative of a primary infection. However, we do not expect any misclassification to be related to toxic metal exposures and thus should not have biased our findings. Finally, only individuals aged 6–49 years with surplus sera were eligible for CMV testing in the 1999–2004 NHANES cycles. These inclusion criteria reduced the statistical power of our analyses and additionally precluded evaluations of the very young and of older adults, for whom associations of toxic metals exposures and CMV may differ.

In summary, this novel study addresses a need to improve the current understanding of the immune-related health effects of environmental exposures and how life stage may influence susceptibility. Using data from a nationally-representative sample of the United States population, we observed nonsignificant associations between blood cadmium concentrations with CMV infection, CMV recurrence, and CMV-specific IgG antibody levels. The evidence for environmentally-relevant levels of lead exposure was mixed. We found null associations in children and older adults but observed higher blood lead levels to be associated with CMV seropositivity and antibody levels in young adults, which may have public health implications for mother-to-child viral transmission of congenital infections as well as for long-term chronic disease risk. Future studies are needed to confirm our findings. Moreover, research in the field of environmental epidemiology should consider the impact of toxic metal exposures on innate and adaptive immune responses, and how these relationships might contribute to both infectious and chronic diseases.

## Supplementary Material


