# Supplementary material for: Cytomegalovirus seroprevalence, recurrence, and antibody levels: Associations with cadmium and lead exposures in the general United States population
Source: Environ Epidemiol. 2020 Jun 5;4(4):e100. doi: 10.1097/EE9.0000000000000100 (PMC7423529; doi:10.1097/EE9.0000000000000100)
Supplement: Supplementary file 1 [file ee9-4-e0100-s001.docx]

| **Supplemental Table 1. CMV infection classification by IgG, IgM, shedding, and IgG avidity among individuals aged 6-49 years, NHANES 1999-2004** | | | | | |
| --- | --- | --- | --- | --- | --- |
| **CMV Classification** | **IgG** | **IgM** | **Shedding** | **IgG Avidity** | **Unweighted N** |
| Seronegative | Negative | Missing | Missing | Missing | 5,990 |
| Primary infection | Positive | Positive | Positive | Low | 14 |
|  | Positive | Positive | Negative | Low | 10 |
|  | Positive | Negative | Positive | Low | 8 |
|  | Positive | Positive | Missing | Low | 2 |
|  |  |  |  |  | 34 (total) |
| Latent infection | Positive | Negative | Negative | Missing | 5,418 |
|  | Positive | Negative | Negative | Low | 156 |
|  | Positive | Negative | Negative | High | 4 |
|  |  |  |  |  | 5,578 (total) |
| Recurrent infection | Positive | Negative | Positive | High | 152 |
|  | Positive | Positive | Negative | High | 46 |
|  | Positive | Positive | Positive | High | 16 |
|  | Positive | Positive | Missing | High | 10 |
|  |  |  |  |  | 224 (total) |

| **Supplemental Table 2. Summary of laboratory methods used to determine CMV IgG and IgM seropositivity, detect CMV DNA in urine, and measure CMV IgG avidity in NHANES 1999-2004.** | | | | | |
| --- | --- | --- | --- | --- | --- |
| **CMV Parameter** | **Specimen** | **Testing Method** | **Interpretation** | **Eligible Sample** | **Unweighted N (Weighted %)** |
| IgG | Sera | Commercial ELISA (Quest International, Inc.) | Positive if optical density values exceeded the manufacturer’s suggested cutoff value.  Note: For a small number of samples near the cutoff, a second commercial ELISA (bioMérieux Inc.) was performed with specimens classified as positive if optical density values from both ELISAs exceeded the respective manufacturers’ suggested cutoff values. If these two tests disagreed, a commercial IFA (Bion International, Inc.) was performed with fluorescent intensities exceeding a cutoff value reported as positive. | Participants aged 6 to 49 years with stored sera | 7,698 positive of 13,688 eligible (50.8%) |
| IgM | Sera | Commercial ELISA (Diamedix) | Optical density ratios <0.90 as negative, ≥0.90 to <1.10 as equivocal, and ≥1.10 as positive.  Note: Confirmatory testing was performed on specimens near the test cutoff values using the commercial Vidas ELISA test (bioMerieux, Inc.) with results interpreted according to the manufacturers’ instructions. | Participants who were CMV IgG seropositive with stored sera | 101 positive of 7,070 eligible (1.9%) |
| IgG avidity | Sera | Commercial Vidas ELISA (bioMerieux, Inc.) | Avidity indexes ≥ 0.8 were interpreted as high avidity (suggestive of past infection), >0.7 and <0.8 as indeterminate, and <0.7 as low avidity (suggestive of recent infection). | 1. Participants who were CMV IgG and IgM seropositive with stored sera 2. A similar number of randomly selected CMV IgG seropositive and IgM seronegative participants with stored sera | 38 low avidity of 418 eligible (10.8%) |
| CMV shedding | Urine | RT-PCR | PCR testing was performed in duplicate for all specimens, with two positive results required for specimens to be reported as positive. | Participants who were CMV IgG seropositive with stored urine | 483 shedding of 6,129 eligible (5.3%) |

| **Supplemental Table 3. Prevalence ratios (95% CI) of CMV IgG seropositivity by toxic metal biomarker and age group, NHANES 1999-2004 (N=13,688)** | | | | | | | | | | | | | | | | | | |
| --- | --- | --- | --- | --- | --- | --- | --- | --- | --- | --- | --- | --- | --- | --- | --- | --- | --- | --- |
|  |  | **6-19 years (N = 7,465)** | | |  | **20-29 years (N = 2,113)** | | | | |  | | **30-49 years (N = 4,110)** | | | | | |
| **Blood cadmium** | **Unweighted**  **N** | **Unadjusted** | **Minimally adjusted^a^** | **Fully adjusted^b^** |  | **Unweighted**  **N** | **Unadjusted** | **Minimally**  **adjusted^a^** | **Fully adjusted^b^** |  | | **Unweighted**  **N** | | **Unadjusted** | **Minimally adjusted^a^** | **Fully adjusted^b^** | ***P_interaction_*^b^** |  |
| **<0.30 μg/L^c^** | 4,695 | 1.00  (reference) | 1.00  (reference) | 1.00  (reference) |  | 821 | 1.00  (reference) | 1.00  (reference) | 1.00  (reference) |  | | 1,223 | | 1.00  (reference) | 1.00  (reference) | 1.00  (reference) | 0.36 |  |
| **0.21-0.30 μg/L** | 1,184 | 1.11  (1.01-1.22) | 1.04  (0.94-1.15) | 1.04  (0.94-1.15) |  | 365 | 1.12  (0.93-1.34) | 1.03  (0.89-1.29) | 1.03  (0.89-1.20) |  | | 606 | | 1.08  (0.94-1.23) | 0.97  (0.85-1.10) | 0.96  (0.95-1.09) |  |  |
| **0.31-0.50 μg/L** | 1,095 | 1.16  (1.04-1.30) | 1.02  (0.90-1.15) | 1.02  (0.90-1.15) |  | 473 | 1.27  (1.09-1.48) | 1.13  (1.00-1.29) | 1.14  (1.00-1.30) |  | | 1,009 | | 1.27  (1.13-1.42) | 1.07  (0.95-1.19) | 1.06  (0.95-1.19) |  |  |
| **0.51-8.50 μg/L** | 491 | 1.09  (0.91-1.29) | 1.09  (0.87-1.35) | 1.09  (0.87-1.35) |  | 454 | 1.01  (0.86-1.19) | 1.03  (0.86-1.24) | 1.03  (0.86-1.24) |  | | 1,272 | | 1.30  (1.18-1.44) | 1.08  (0.97-1.21) | 1.08  (0.97-1.20) |  |  |
| ***P_trend_*** |  | 0.03 | 0.47 | 0.46 |  |  | 0.47 | 0.39 | 0.36 |  | |  | | <0.01 | 0.11 | 0.12 |  |  |
| **Blood lead** | **Unweighted**  **N** | **Unadjusted** | **Minimally adjusted^a^** | **Fully adjusted^b^** |  | **Unweighted**  **N** | **Unadjusted** | **Minimally adjusted^a^** | **Fully adjusted^b^** |  | | **Unweighted**  **N** | | **Unadjusted** | **Minimally adjusted^a^** | **Fully adjusted^b^** | ***P_interaction_*^b^** |  |
| **<0.80 μg/dL** | 1,672 | 1.00  (reference) | 1.00  (reference) | 1.00  (reference) |  | 580 | 1.00  (reference) | 1.00  (reference) | 1.00  (reference) |  | | 506 | | 1.00  (reference) | 1.00  (reference) | 1.00  (reference) | 0.06 |  |
| **0.81-1.20 μg/dL** | 2,559 | 1.08  (0.96-1.21) | 1.06  (0.93-1.19) | 1.06  (0.93-1.19) |  | 707 | 1.05  (0.88-1.26) | 1.03  (0.86-1.23) | 1.03  (0.86-1.23) |  | | 1,007 | | 0.89  (0.78-1.02) | 0.93  (0.81-1.08) | 0.94  (0.81-1.08) |  |  |
| **1.21-1.89 μg/dL** | 1,621 | 1.22  (1.09-1.38) | 1.12  (0.99-1.27) | 1.12  (0.99-1.27) |  | 398 | 1.27  (1.04-1.56) | 1.23  (1.02-1.48) | 1.23  (1.03-1.48) |  | | 1,055 | | 0.97  (0.85-1.11) | 1.01  (0.88-1.15) | 1.01  (0.89-1.16) |  |  |
| **1.90-68.90 μg/dL** | 1,613 | 1.28  (1.14-1.45) | 1.08  (0.96-1.21) | 1.08  (0.96-1.21) |  | 428 | 1.58  (1.34-1.86) | 1.25  (1.04-1.52) | 1.25  (1.04-1.51) |  | | 1,542 | | 0.95  (0.83-1.09) | 1.00  (0.87-1.15) | 1.00  (0.87-1.16) |  |  |
| ***P_trend_*** |  | <0.01 | 0.10 | 0.10 |  |  | <0.01 | <0.01 | <0.01 |  | |  | | 0.96 | 0.48 | 0.46 |  |  |
| ^a^ Adjusted for age (years, continuous), gender, race/ethnicity (non-Hispanic White, non-Hispanic Black, Mexican American, other Hispanic, other race), education (less than high school diploma, high school diploma or equivalent, more than high school), family income: poverty line (continuous), nativity (born in the 50 U.S. or elsewhere), and serum cotinine (ng/mL, continuous) with mutual adjustment for blood cadmium and lead categories. Prevalence ratios were weighted to account for the complex survey design and 95% confidence intervals were estimated using Taylor series linearization.  ^b^ Adjusted for all variables listed above, in addition to crowding index (residents per room, continuous)  **^c^** Below the highest limit of detection across NHANES cycles 1999-2000, 2001-2002, and 2003-2004 | | | | | | | | | | | | | | | | | |  |

| **Supplemental Table 4. Prevalence ratios (95% CI) of CMV recurrence by toxic metal biomarker, NHANES 1999-2004 (N=5,802)** | | | | | |  |
| --- | --- | --- | --- | --- | --- | --- |
| **Blood cadmium** | **Unweighted N** | **Unadjusted** | **Minimally adjusted^a^** | **Fully adjusted^b^** |  | |
| **<0.30 μg/L^c^** | 2,598 | 1.00 (reference) | 1.00 (reference) | 1.00 (reference) |  | |
| **0.21-0.30 μg/L** | 912 | 0.73 (0.43-1.24) | 0.83 (0.48-1.45) | 0.83 (0.48-1.45) |  | |
| **0.31-0.50 μg/L** | 1,192 | 0.69 (0.44-1.07) | 0.88 (0.55-1.40) | 0.88 (0.55-1.40) |  | |
| **0.51-8.50 μg/L** | 712 | 0.49 (0.30-0.80) | 0.67 (0.38-1.18) | 0.67 (0.39-1.18) |  | |
| ***P_trend_*** |  | <0.01 | 0.24 | 0.24 |  | |
| **Blood lead** | **Unweighted N** | **Unadjusted** | **Minimally adjusted^a^** | **Fully adjusted^b^** |  | |
| **<0.80 μg/dL** | 1,067 | 1.00 (reference) | 1.00 (reference) | 1.00 (reference) |  | |
| **0.81-1.20 μg/dL** | 1,678 | 1.16 (0.73-1.84) | 1.22 (0.75-1.98) | 1.22 (0.75-1.99) |  | |
| **1.21-1.89 μg/dL** | 1,389 | 0.95 (0.53-1.71) | 1.25 (0.64-2.43) | 1.24 (0.64-2.43) |  | |
| **1.90-68.90 μg/dL** | 1,668 | 0.60 (0.34-1.06) | 0.93 (0.43-2.03) | 0.93 (0.43-2.03) |  | |
| ***P_trend_*** |  | 0.04 | 0.92 | 0.91 |  | |
| ^a^ Adjusted for age (years, continuous), gender, race/ethnicity (non-Hispanic White, non-Hispanic Black, Mexican American, other Hispanic, other race), education (less than high school diploma, high school diploma or equivalent, more than high school), family income: poverty line (continuous), nativity (born in the 50 U.S. or elsewhere), and serum cotinine (ng/mL, continuous) with mutual adjustment for blood cadmium and lead categories. Prevalence ratios were weighted to account for the complex survey design and 95% confidence intervals were estimated using Taylor series linearization.  ^b^ Adjusted for all variables listed above, in addition to crowding index (residents per room, continuous)  **^c^** Below the highest limit of detection across NHANES cycles 1999-2000, 2001-2002, and 2003-2004 | | | | | |  |

| **Supplemental Table 5. Mean differences (95% CI) in CMV IgG ELISA optical density values by toxic metal biomarker and age group, NHANES 1999-2004 (N=5,802)** | | | | | | | | | | | | | | | | |
| --- | --- | --- | --- | --- | --- | --- | --- | --- | --- | --- | --- | --- | --- | --- | --- | --- |
|  | **6-19 years (N = 2,717)** | | | |  |  | **20-29 years (N = 977)** | |  | | **30-49 years (N = 2,108)** | | | | | |
| **Blood cadmium** | **Unweighted**  **N** | **Unadjusted** | **Minimally**  **adjusted^a^** | **Fully adjusted^b^** |  | **Unweighted**  **N** | **Unadjusted** | **Minimally**  **adjusted^a^** | **Fully adjusted^b^** |  | | **Unweighted**  **N** | **Unadjusted** | **Minimally**  **adjusted^a^** | **Fully adjusted^b^** | ***P_interaction_*^b^** |
| **<0.30 μg/L^c^** | 1,653 | 0.00 (reference) | 0.00 (reference) | 0.00 (reference) |  | 376 | 0.00 (reference) | 0.00 (reference) | 0.00 (reference) |  | | 569 | 0.00 (reference) | 0.00 (reference) | 0.00 (reference) | <0.01 |
| **0.21-0.30 μg/L** | 450 | 0.13 (0.03, 0.24) | 0.09 (-0.01, 0.19) | 0.09 (-0.01, 0.19) |  | 174 | 0.13 (-0.10, 0.36) | 0.10 (-0.11, 0.30) | 0.09 (-0.11, 0.30) |  | | 288 | 0.12 (-0.03, 0.26) | 0.02 (-0.12, 0.17) | 0.03 (-0.12, 0.17) |  |
| **(0.31-0.50 μg/L** | 420 | 0.11 (0.00, 0.22) | 0.07 (-0.05, 0.19) | 0.07 (-0.05, 0.19) |  | 233 | 0.07 (-0.07, 0.21) | -0.01 (-0.15, 0.13) | -0.01 (-0.15, 0.13) |  | | 539 | 0.09 (-0.07, 0.24) | -0.05 (-0.19, 0.08) | -0.05 (-0.19, 0.08) |  |
| **0.51-8.50 μg/L** | 194 | 0.06 (-0.03, 0.16) | 0.06 (-0.09, 0.21) | 0.06 (-0.09, 0.21) |  | 194 | -0.07 (-0.24, 0.10) | -0.08 (-0.30, 0.14) | -0.08 (-0.30, 0.14) |  | | 712 | 0.37 (0.24, 0.50) | 0.05 (-0.10, 0.21) | 0.05 (-0.10, 0.21) |  |
| ***P_trend_*** |  | 0.02 | 0.15 | 0.15 |  |  | 0.50 | 0.45 | 0.46 |  | |  | <0.01 | 0.85 | 0.83 |  |
| **Blood lead** | **Unweighted**  **N** | **Unadjusted** | **Minimally**  **adjusted^a^** | **Fully adjusted^b^** |  | **Unweighted N** | **Unadjusted** | **Minimally**  **adjusted^a^** | **Fully adjusted^b^** |  | | **Unweighted N** | **Unadjusted** | **Minimally**  **adjusted^a^** | **Fully adjusted^b^** | ***P_interaction_*^b^** |
| **<0.80 μg/dL** | 576 | 0.00 (reference) | 0.00 (reference) | 0.00 (reference) |  | 238 | 0.00 (reference) | 0.00 (reference) | 0.00 (reference) |  | | 253 | 0.00 (reference) | 0.00 (reference) | 0.00 (reference) | 0.51 |
| **0.81-1.20 μg/dL** | 907 | -0.02 (-0.13, 0.08) | 0.01 (-0.09, 0.12) | 0.01 (-0.09, 0.12) |  | 287 | -0.07 (-0.25, 0.10) | 0.03 (-0.14, 0.21) | 0.04 (-0.14, 0.21) |  | | 484 | 0.00 (-0.18, 0.18) | 0.06 (-0.12, 0.23) | 0.05 (-0.12, 0.22) |  |
| **1.21-1.89 μg/dL** | 611 | -0.11 (0.20, -0.01) | -0.02 (-0.13, 0.09) | -0.02 (-0.13, 0.09) |  | 203 | -0.01 (-0.20, 0.18) | 0.27 (0.08, 0.46) | 0.27 (0.08, 0.46) |  | | 575 | -0.03 (-0.21, 0.15) | 0.10 (-0.07, 0.27) | 0.10 (-0.08, 0.27) |  |
| **1.90-68.90 μg/dL** | 623 | -0.10 (-0.19, -0.01) | 0.00 (-0.12, 0.11) | 0.00 (-0.12, 0.11) |  | 249 | -0.04 (-0.23, 0.15) | 0.30 (0.08, 0.52) | 0.30 (0.08, 0.52) |  | | 796 | -0.10 (-0.30, 0.09) | 0.09 (-0.12, 0.30) | 0.09 (-0.12, 0.30) |  |
| ***P_trend_*** |  | 0.01 | 0.87 | 0.87 |  |  | 0.81 | <0.01 | <0.01 |  | |  | 0.22 | 0.31 | 0.33 |  |
| ^a^ Adjusted for age (years, continuous), gender, race/ethnicity (non-Hispanic White, non-Hispanic Black, Mexican American, other Hispanic, other race), education (less than high school diploma, high school diploma or equivalent, more than high school), family income: poverty line (continuous), nativity (born in the 50 U.S. or elsewhere), and serum cotinine (ng/mL, continuous) with mutual adjustment for blood cadmium and lead categories. Mean differences were weighted to account for the complex survey design and 95% confidence intervals were estimated using Taylor series linearization.  ^b^ Adjusted for all variables listed above, in addition to crowding index (residents per room, continuous)  **^c^** Below the highest limit of detection across NHANES cycles 1999-2000, 2001-2002, and 2003-2004 | | | | | | | | | | | | | | | | |

**Stata Code**

*****************DATA CLEANING AND SAMPLING WEIGHTS DERIVATION******************

//Import dataset

use "NHANES9904.dta", clear

//Install packages

ssc install groups

ssc install estout

net install gr0002_3, from(http://www.stata-journal.com/software/sj4-3)

ssc install coefplot

//Cleaning + recoding data

*Creating male indicator variable

gen male = .

replace male = 1 if riagendr == 1

replace male = 0 if riagendr == 2

*Health insurance (set refused to answer or don't know to missing)

replace hid010 = . if hid010 == 7 | hid010 == 9

*Number of rooms in house (set refused to answer or don't know to missing)

replace hod050 = . if hod050 == 777 | hod050 == 999

*Education

gen education = .

replace education = dmdhredu if ridageyr < 25 // use household reference person

replace education = dmdeduc2 if ridageyr >= 25 // use participant

recode education 7 = . // refused to missing

recode education 9 = . // don't know to missing

recode education 2 = 1 // collapse w/ less than 9th grade

recode education 5 = 4 // collapse w/ some college

*US Foreign

gen us_foreign = .

replace us_foreign = 0 if dmdborn == 1

replace us_foreign = 1 if dmdborn == 2 | dmdborn == 3

*Household crowding index

gen crowding = dmdhhsiz/hod050

*Limits of Detection

*For lead, the LOD across all cycles was 0.3 ug/dL

*For cadmium, the LOD for cycles 1999-2002 was 0.3 ug/L, for 2003-2004,

* it was 0.2 ug/L;

*For cotinine, the detection limits in each two year cycle from 1999 to 2002 has changed.

*For 1999-2000 the detection limit was .05 and the below the limit of detection value was .035.

*For 2001-2002 there were two detection limits and below the limit of detection values.

*One of the detection limits was .05 and the below the limit of detection value was .035.

*The other detection limit was .015 and the below the limit of detection value was .011.;

*For 2003-2004 the detection limit was .015 and the below the limit of detection value was .011.;

*Flag values < LOD - use the highest across the 3 cycles:

gen cadmium_lod = .

replace cadmium_lod = 0 if !missing(lbxbcd) & lbxbcd >= 0.3

replace cadmium_lod = 1 if !missing(lbxbcd) & lbxbcd < 0.3

gen lead_lod = .

replace lead_lod = 0 if !missing(lbxbpb) & lbxbpb >= 0.3

replace lead_lod = 1 if !missing(lbxbpb) & lbxbpb < 0.3

gen cotinine_lod = 0

replace cotinine_lod = 1 if !missing(lbxcot) & lbxcot < 0.05

replace cotinine_lod = . if missing(lbxcot)

*Rename variables

gen cadmium = lbxbcd

gen lead = lbxbpb

gen cotinine = lbxcot

*Rescale the MEC weights since combining 3 cycles

gen wtmec6yr = wtmec4yr*(2/3) if sddsrvyr == 1 | sddsrvyr == 2 // NHANES 1999-2002

replace wtmec6yr = wtmec2yr*(1/3) if sddsrvyr == 3 // NHANES 2003-2004

*Create 3 age groups (6-11, 12-19, and 20-49 years)

gen agegroup = .

replace agegroup = 1 if ridageyr >= 6 & ridageyr <= 11

replace agegroup = 2 if ridageyr >= 12 & ridageyr <= 19

replace agegroup = 3 if ridageyr >= 20 & ridageyr <= 49

*Create 30 age/sex/racial groups

egen agesexracegroup = group(agegroup ridreth1 male)

*Create a flag for participants with stored surplus sera or urine available for CMV testing

gen cmvspecimenflag = 0

replace cmvspecimenflag = 1 if !missing(sscmvod) | !missing(ssucysh)

*Save NHANES dataset

save "/NHANES9904.dta", replace

*Calculate weighted proportion of available CMV serum samples for each age/sex/racial group (n=30)

//setting sampling weights, clustering and strata variables

svyset sdmvpsu[pweight=wtmec6yr], strata(sdmvstra)

forval i = 1/30 {

qui estpost svy, subpop(if agesexracegroup==`i'): tab cmvspecimenflag

qui esttab ., cell("b(f(4))") nostar noobs nonumber not nomtitle collabel(none)

mat percent=e(b)

di percent[1,2]

eststo clear

}

*Input proportions of non-missing CMV serum data

clear

input wtcmvserum

.76027739

.79762496

.64997402

.59082428

.6709199

.74329468

.69178479

.73495199

.56838764

.77577281

.87601133

.87540671

.84669303

.88244396

.86359995

.85486537

.87043562

.88353762

.79554003

.83957656

.93693979

.91514309

.92425305

.9639366

.93243411

.94261215

.89807375

.90189461

.89269602

.95091689

end

gen agesexracegroup = _n

*Merge with NHANES dataset

merge 1:m agesexracegroup using "NHANES9904.dta"

drop _merge

*Re-weight MEC weights:

gen wtmec6yr_cmvs = wtmec6yr*wtcmvserum

*Create new variables

gen cmv_igg = .

replace cmv_igg = 0 if sscmv == 2

replace cmv_igg = 1 if sscmv == 1

gen cmv_igm = .

replace cmv_igm = 0 if sscmigm == 2

replace cmv_igm = 1 if sscmigm == 1

gen lowavidity = .

replace lowavidity = 0 if sscmigga == 2

replace lowavidity = 1 if sscmigga == 1

gen shedding = .

replace shedding = 0 if ssucysh == 2

replace shedding = 1 if ssucysh == 1

gen viralload = ssuctvl

replace viralload = 80 if missing(ssuctvl) & !missing(shedding)

*Eligiblity

gen eligible = 0

replace eligible = 1 if lbdhi != 1 & !missing(agegroup) & !missing(sscmv) & sscmv != 3 & ///

!missing(lbxbcd) & !missing(lbxbpb) & !missing(education) & !missing(indfmpir) & ///

dmdborn != 7 & dmdborn != 9 & !missing(lbxcot) & !missing(hod050) & hod050 != 777 & ///

hod050 != 9999 & !missing(wtmec6yr_cmvs) & wtmec6yr_cmvs != 0

egen cmv_category = group(cmv_igg sscmigm ssucysh sscmigga)

groups cmv_category cmv_igg sscmigm ssucysh sscmigga if eligible==1, missing

replace cmv_category = 0 if cmv_igg == 0 // not infected (susceptible)

*1 = primary; 2 = recurrence; 3 = latent;

recode cmv_category 3 = . // active but missing low avidity (n=3)

recode cmv_category 4 = 1 // primary (n=10)

recode cmv_category 5 = 2 // recurrence (n=46)

recode cmv_category 6 = 1 // primary (n=1)

recode cmv_category 7 = 1 // primary (n=8)

recode cmv_category 8 = 2 // recurrence (n=152)

recode cmv_category 9 = . // active but equivocal low avidity (n=2)

recode cmv_category 10 = . // active but missing low avidity (n=283)

recode cmv_category 11 = 3 // latent (n=4)

recode cmv_category 12 = 3 // latent (n=156)

recode cmv_category 13 = 3 // latent (n=2)

recode cmv_category 14 = 3 // latent (n=5,416)

recode cmv_category 15 = . // not producing IgM, equivocal urine CMV PCR (n=23)

recode cmv_category 16 = . // missing low avidity (n=4)

recode cmv_category 17 = . // equivocal CMV IgM serology, not shedding (n=6)

recode cmv_category 18 = . // equivocal CMV IgM serology, equivocal PCR (n=1)

replace cmv_category = 1 if cmv_igg == 1 & sscmigm==1 & missing(ssucysh) & sscmigga==1 // primary (n=1)

replace cmv_category = 2 if cmv_igg==1 & sscmigm==1 & missing(ssucysh) & sscmigga==2 // recurrence (n=10)

gen cmv_reac = .

replace cmv_reac = 0 if cmv_category == 3 // latent

replace cmv_reac = 1 if cmv_category == 2 // recurrence

*Create new age groups (6-11, 12-19, 20-29, 30-39, 40-49 years)

drop agegroup

gen agegroup = .

replace agegroup = 1 if ridageyr >= 6 & ridageyr <= 11

replace agegroup = 2 if ridageyr >= 12 & ridageyr <= 19

replace agegroup = 3 if ridageyr >= 20 & ridageyr <= 29

replace agegroup = 4 if ridageyr >= 30 & ridageyr <= 39

replace agegroup = 5 if ridageyr >= 40 & ridageyr <= 49

*Label variables

label define male 1 "Male" 0 "Female"

label values male male

label define us_native 0 "Born in US territory or other country" 1 "Born in 50 US States or Washington, DC"

label values us_native us_native

label define us_foreign 0 "Born in 50 US States or Washington, DC" 1 "Born in US territory or other country"

label values us_foreign us_foreign

label define ridreth1 1 "Mexican American" 2 "Other Hispanic" 3 "Non-Hispanic white" 4 "Non-Hispanic black" 5 "Other Race - Including Multi-Racial"

label values ridreth1 ridreth1

label define education 1 "Less than high school" 3 "High school" 4 "More than high school"

label values education education

*Selection criteria for CMV IgG serostatus analyes

gen sel_1 = 0

replace sel_1 = 1 if eligible==1 & !missing(cmv_igg)

*Selection criteria for CMV recurrence analyses

gen sel_2 = 0

replace sel_2 = 1 if sel_1==1 & !missing(cmv_reac)

*Create quartiles for blood cadmium, blood lead, and urine cadmium

_pctile cadmium if sel_1==1 & cadmium_lod==0 [pweight=wtmec6yr_cmvs], p(33.33 66.67)

return list

gen cadmium_q = .

replace cadmium_q = 1 if cadmium < 0.3 // below highest LOD

replace cadmium_q = 2 if cadmium >= 0.3 & cadmium < 0.4000000059604645

replace cadmium_q = 3 if cadmium >= 0.4000000059604645 & cadmium < 0.6000000238418579

replace cadmium_q = 4 if cadmium >= 0.6000000238418579 & !missing(cadmium)

summarize cadmium

_pctile lead if sel_1==1 [pweight=wtmec6yr_cmvs], p(25 50 75)

return list

gen lead_q = .

replace lead_q = 1 if !missing(lead) & lead < 0.800000011920929

replace lead_q = 2 if !missing(lead) & lead >= 0.800000011920929 & lead < 1.200000047683716

replace lead_q = 3 if !missing(lead) & lead >= 1.200000047683716 & lead < 1.899999976158142

replace lead_q = 4 if !missing(lead) & lead >= 1.899999976158142

summarize lead

_pctile urxucd if sel_1==1 [pweight=wtmec6yr_cmvs], p(25 50 75)

return list

gen ucadmium_q = .

replace ucadmium_q = 1 if !missing(urxucd) & urxucd < 0.09

replace ucadmium_q = 2 if !missing(urxucd) & urxucd >= 0.09 & urxucd < 0.18

replace ucadmium_q = 3 if !missing(urxucd) & urxucd >= 0.18 & urxucd < 0.35

replace ucadmium_q = 4 if !missing(urxucd) & urxucd >= 0.35

summarize urxucd

*Re-define age groups

gen agegroup_3 = .

replace agegroup_3 = 1 if ridageyr >= 6 & ridageyr <= 19

replace agegroup_3 = 2 if ridageyr >= 20 & ridageyr <= 29

replace agegroup_3 = 3 if ridageyr >= 30 & ridageyr <= 49

*Save NHANES dataset

save “NHANES9904.dta", replace

*****************ANALYSIS******************

//Survey set the data

svyset [w=wtmec6yr_cmvs], psu(sdmvpsu) strata(sdmvstra)

//CMV seroprevalence

svy, subpop(if sel_1==1): tab sscmv, percent ci // 50.8% (48.3-55.4%)

//Table 1

foreach v of varlist agegroup_1 agegroup_3 education us_foreign {

svy, subpop(if sel_1==1): tab `v' cmv_igg, col percent ci

svy, subpop(if sel_2==1): tab `v' cmv_reac, col percent ci

}

foreach v of varlist indfmpir crowding {

svy, subpop(if sel_1==1 & cmv_igg==0): mean `v'

svy, subpop(if sel_1==1 & cmv_igg==1): mean `v'

svy, subpop(if sel_2==1 & cmv_reac==0): mean `v'

svy, subpop(if sel_2==1 & cmv_reac==1): mean `v'

}

foreach v of varlist cotinine_ln cadmium_ln lead_ln {

qui svy, subpop(if sel_1==1 & cmv_igg==0): mean `v'

nlcom exp(_b[`v'])

qui svy, subpop(if sel_1==1 & cmv_igg==1): mean `v'

nlcom exp(_b[`v'])

qui svy, subpop(if sel_2==1 & cmv_reac==0): mean `v'

nlcom exp(_b[`v'])

qui svy, subpop(if sel_2==1 & cmv_reac==1): mean `v'

nlcom exp(_b[`v'])

}

svy, subpop(if sel_1==1 & cmv_igg==1): tobit sscmvod, ul(3.001)

svy, subpop(if sel_2==1 & cmv_reac==0): tobit sscmvod, ul(3.001)

svy, subpop(if sel_2==1 & cmv_reac==1): tobit sscmvod, ul(3.001)

svy, subpop(if sel_1==1): tab shedding cmv_igg, col percent ci

svy, subpop(if sel_2==1): tab shedding cmv_reac, col percent ci

*Proportion < LOD

svy, subpop(if sel_1==1): tab cadmium_lod

svy, subpop(if sel_1==1): tab lead_lod

svy, subpop(if sel_1==1): tab cotinine_lod

*Correlations between cadmium and lead:

egen pb_rank = rank(lead)

egen cd_rank = rank(cadmium)

pwcorr pb_rank cd_rank if sel_1==1 [aweight=wtmec6yr_cmvs], sig

//Regression models

global minimum c.ridageyr i.ridreth1 i.male c.cotinine c.indfmpir i.education i.us_foreign

global full c.ridageyr i.ridreth1 i.male c.cotinine c.indfmpir i.education i.us_foreign c.crowding

*****************CMV Seropositivity******************

//Cadmium x Lead interaction assessment

forval i = 1/3 {

qui svy, subpop(if sel_1==1 & agegroup_3==`i'): poisson cmv_igg c.cadmium_q##c.lead_q $full, eform

testparm c.cadmium_q#c.lead_q

}

//Cadmium/Lead x Age interaction assessment

qui svy, subpop(if sel_1==1): poisson cmv_igg i.cadmium_q##i.agegroup_3 i.lead_q##i.agegroup_3 $full, eform

testparm cadmium_q#agegroup_3

testparm lead_q#agegroup_3

//Final seroprevalence models - stratified by age 6-19 years, 20-29 years, 30-49 years

forval i = 1/3 {

svy, subpop(if sel_1==1 & agegroup_3==`i'): poisson cmv_igg i.cadmium_q i.lead_q, eform

qui svy, subpop(if sel_1==1 & agegroup_3==`i'): poisson cmv_igg c.cadmium_q c.lead_q, eform

testparm cadmium_q

testparm lead_q

svy, subpop(if sel_1==1 & agegroup_3==`i'): poisson cmv_igg i.cadmium_q i.lead_q $minimum, eform

qui svy, subpop(if sel_1==1 & agegroup_3==`i'): poisson cmv_igg c.cadmium_q c.lead_q $minimum, eform

testparm cadmium_q

testparm lead_q

svy, subpop(if sel_1==1 & agegroup_3==`i'): poisson cmv_igg i.cadmium_q i.lead_q $full, eform

estimates store cmv_igg_`i'

qui svy, subpop(if sel_1==1 & agegroup_3==`i'): poisson cmv_igg c.cadmium_q c.lead_q $full, eform

testparm cadmium_q

testparm lead_q

}

*****************CMV IgG Levels******************

//Cadmium x Lead interaction assessment

forval i = 1/3 {

qui svy, subpop(if sel_2==1 & agegroup_3==`i'): tobit sscmvod c.cadmium_q##c.lead_q $full, ul(3.001)

testparm c.cadmium_q#c.lead_q

}

//Cadmium/Lead x Age interaction assessment

qui svy, subpop(if sel_2==1): tobit sscmvod i.cadmium_q##i.agegroup_3 i.lead_q##i.agegroup_3 $full, ul(3.001)

testparm cadmium_q#agegroup_3

testparm lead_q#ageagroup_3

//Final CMV IgG level models - stratified by age 6-19 years, 20-29 years, 30-49 years

forval i = 1/3 {

svy, subpop(if sel_2==1 & agegroup_3==`i'): tobit sscmvod i.cadmium_q i.lead_q, ul(3.001)

qui svy, subpop(if sel_2==1 & cmv_igm==0 & agegroup_3==`i'): tobit sscmvod c.cadmium_q c.lead_q, ul(3.001)

testparm cadmium_q

testparm lead_q

svy, subpop(if sel_2==1 & agegroup_3==`i'): tobit sscmvod i.cadmium_q i.lead_q $minimum, ul(3.001)

qui svy, subpop(if sel_2==1 & agegroup_3==`i'): tobit sscmvod c.cadmium_q c.lead_q $minimum, ul(3.001)

testparm cadmium_q

testparm lead_q

svy, subpop(if sel_2==1 & agegroup_3==`i'): tobit sscmvod i.cadmium_q i.lead_q $full, ul(3.001)

estimates store igg_level_`i'

qui svy, subpop(if sel_2==1 & agegroup_3==`i'): tobit sscmvod c.cadmium_q c.lead_q $full, ul(3.001)

testparm cadmium_q

testparm lead_q

}

*****************CMV Recurrence******************

//Cadmium x Lead interaction assessment

forval i = 1/3 {

qui svy, subpop(if sel_2==1 & agegroup_3==`i'): poisson cmv_reac c.cadmium_q##c.lead_q $full, eform

testparm c.cadmium_q#c.lead_q

}

//Cadmium/Lead x Age interaction assessment

qui svy, subpop(if sel_2==1): poisson cmv_reac i.cadmium_q##i.agegroup_3 i.lead_q##i.agegroup_3 $full, eform

testparm cadmium_q#agegroup_3

testparm lead_q#agegroup_3

//Final CMV recurrence models - not age-stratified

svy, subpop(if sel_2==1): poisson cmv_reac i.cadmium_q i.lead_q, eform

qui svy, subpop(if sel_2==1): poisson cmv_reac c.cadmium_q c.lead_q, eform

testparm cadmium_q

testparm lead_q

svy, subpop(if sel_2==1): poisson cmv_reac i.cadmium_q i.lead_q $minimum, eform

qui svy, subpop(if sel_2==1): poisson cmv_reac c.cadmium_q c.lead_q $minimum, eform

testparm cadmium_q

testparm lead_q

svy, subpop(if sel_2==1): poisson cmv_reac i.cadmium_q i.lead_q $full, eform

estimates store cmv_reac

qui svy, subpop(if sel_2==1): poisson cmv_reac c.cadmium_q c.lead_q $full, eform

testparm cadmium_q

testparm lead_q

*****************Sensitivity Analysis: Urinary Cadmium******************

//Final seroprevalence models - stratified by age 6-19 years, 20-29 years, 30-49 years

forval i = 1/3 {

svy, subpop(if sel_1==1 & agegroup_3==`i'): poisson cmv_igg i.ucadmium_q i.lead_q, eform

qui svy, subpop(if sel_1==1 & agegroup_3==`i'): poisson cmv_igg c.ucadmium_q c.lead_q, eform

testparm ucadmium_q

testparm lead_q

svy, subpop(if sel_1==1 & agegroup_3==`i'): poisson cmv_igg i.ucadmium_q i.lead_q $minimum urxucr, eform

qui svy, subpop(if sel_1==1 & agegroup_3==`i'): poisson cmv_igg c.ucadmium_q c.lead_q $minimum urxucr, eform

testparm ucadmium_q

testparm lead_q

svy, subpop(if sel_1==1 & agegroup_3==`i'): poisson cmv_igg i.ucadmium_q i.lead_q $full urxucr, eform

qui svy, subpop(if sel_1==1 & agegroup_3==`i'): poisson cmv_igg c.ucadmium_q c.lead_q $full urxucr, eform

testparm ucadmium_q

testparm lead_q

}
